# Factors associated with pulmonary embolism in children with refractory *Mycoplasma pneumoniae* pneumonia and elevated D-dimer

**DOI:** 10.3389/fped.2026.1847107

**Published:** 2026-06-25

**Authors:** Geng Wang, Luanjie Yao, Bao Tie, Muguoer Wang, Xuemei Bai, Jing Li, Yong Feng, Zhaorui Yang, Haojing Tang, Li Chen, Si Liu, Bing Dai, Yunxiao Shang, Jichun Wang, Ning Chen

**Affiliations:** 1 Department of Pediatrics, Shengjing Hospital of China Medical University, Shenyang, Liaoning, China; 2 Department of Pediatrics, The Affiliated Hospital of Inner Mongolia Medical University, Hohhot, Inner Mongolia, China; 3 Department of Pediatrics, Inner Mongolia People’s Hospital, Hohhot, Inner Mongolia, China; 4 Department of Pediatrics, Dalian University of Technology Affiliated Central Hospital, Dalian, Liaoning, China; 5 Department of Pediatrics, The First Affiliated Hospital of Jinzhou Medical University, Jinzhou, China

**Keywords:** autoantibody, children, d-dimer, pulmonary embolism, refractory *mycoplasma pneumoniae* pneumonia

## Abstract

**Objective:**

Pulmonary embolism (PE) is a serious complication of refractory *Mycoplasma pneumoniae* pneumonia (RMPP), and its diagnosis is often delayed because of nonspecific clinical presentations. This study aimed to investigate factors associated with PE in children with RMPP and elevated D-dimer.

**Methods:**

Children diagnosed with RMPP and suspected PE were enrolled from five hospitals in northern China between November 2023 and March 2024. Blood samples were collected for inflammatory and immunological assessments. Patients were stratified into PE and non-PE groups according to computed tomographic pulmonary angiography findings. Univariate and multivariate logistic regression analyses were performed to identify factors associated with PE.

**Results:**

Overall, 109 children with RMPP were included, and 49 (45.0%) had PE. Clinical features did not differ significantly between the PE and non-PE groups (*P* > 0.05). Compared with the non-PE group, children with PE had higher white blood cell and neutrophil counts and higher admission and peak D-dimer levels, whereas admission CRP, LDH, ferritin, and IL-6 levels were comparable between groups. Simultaneous positivity for lupus anticoagulant and antinuclear antibodies (*P* = 0.006) and left lower lobe consolidation (*P* = 0.002) were also more frequent in the PE group. In multivariate logistic regression analysis, neutrophil count (OR, 1.221; 95% CI, 1.031–1.446), log_2_(admission D-dimer/500 μg/L) (OR, 2.664; 95% CI, 1.485–4.778), simultaneous positivity for lupus anticoagulant and antinuclear antibodies (OR, 3.185; 95% CI, 1.234–8.223), and left lower lobe consolidation (OR, 5.016; 95% CI, 1.825–13.790) were independently associated with PE.

**Conclusions:**

Among children with RMPP, elevated D-dimer levels, and clinical suspicion of PE who underwent CTPA, higher neutrophil counts, greater D-dimer elevation, simultaneous positivity for lupus anticoagulant and antinuclear antibodies, and left lower lobe consolidation were independently associated with PE.

## 1 Introduction

*Mycoplasma pneumoniae (M. pneumoniae)* is a prevalent etiological agent of community-acquired pneumonia in children, especially those aged ≥5 years (1, 2). Although *M. pneumoniae* pneumonia (MPP) is usually a self-limiting condition, certain cases exhibit persistent fever and clinical deterioration despite the administration of appropriate antibiotic therapy, a condition referred to as refractory MPP (RMPP) (3, 4). The predominant mechanism underlying RMPP is believed to be an excessive inflammatory response, which can lead to severe cases and a range of extrapulmonary complications (4). Pulmonary embolism (PE), as one of the serious complications, has been increasingly reported over the past decade (5–12). The diagnosis of PE is frequently delayed, often occurring weeks to a few months after disease onset (5, 9, 11–13), and 57% of patients are initially diagnosed with other respiratory diseases (13). This delay is primarily attributable to the nonspecific clinical presentations of PE, such as tachycardia, dyspnea, chest pain, hypoxemia, and hemoptysis, which closely resemble the manifestations of MPP. Computed tomography pulmonary angiography (CTPA) is widely acknowledged as the gold standard for diagnosing PE (14). However, its invasive nature and associated potential risks preclude its routine application in pediatric populations. Consequently, determining the optimal strategy for early identification of PE in children with MPP is warranted.

Several retrospective studies have attempted to investigate the risk factors for PE in children with MPP (9, 10, 12). Factors such as age, D-dimer and C-reactive protein (CRP) levels, lobar consolidation, and pleural effusion have been posited as potential associations with PE in the context of MPP. However, the findings have been inconsistent, primarily due to the retrospective nature of the studies and variations in the inclusion criteria, including MPP severity, PE symptoms, and D-dimer levels. D-dimer, a degradation product of cross-linked fibrin, has been recommended as a diagnostic tool for ruling out PE because of its high negative predictive value (14–16). However, D-dimer testing lacks utility for confirming PE, as elevated D-dimer levels can also be observed in patients with malignancies, inflammatory disorders, and severe infections (14). Elevated D-dimer levels, along with increased CRP levels and lobar consolidation, indicate an excessive inflammatory response in RMPP (17, 18). Whether the identified risk factors are attributable to RMPP or its associated PE remains unclear. Additionally, transient production of lupus anticoagulants (LAC) (5, 19) and antinuclear antibodies (ANA) (5, 9, 11) has been observed in a limited number of patients with MPP and thrombosis who underwent autoantibody tests. LAC and ANA have been reported to be associated with thrombotic events in autoimmune diseases, possibly through endothelial dysfunction and hypercoagulability (20, 21). Therefore, we hypothesized that these factors could serve as potential factors associated with PE in children with RMPP. This study aimed to validate previously reported factors associated with PE and to investigate whether LAC and ANA were associated with the occurrence of PE in children with RMPP.

## 2 Materials and methods

### 2.1 Study design and participants

Children diagnosed with RMPP and suspected PE were enrolled from five hospitals in northern China during the period of November 2023 to March 2024. Inclusion criteria for patients were as follows: 1) Age < 14 years; 2) Confirmed diagnosis of RMPP; 3) Suspected PE, defined as D-dimer levels ≥500 μg/L together with at least one clinical manifestation suggestive of PE, such as tachycardia, dyspnea, shortness of breath, chest pain, hypoxemia, or hemoptysis, or D-dimer levels ≥1000 μg/L regardless of symptoms (14). The exclusion criteria were as follows: 1) Positive results for respiratory syncytial virus, adenovirus, or influenza virus A and B polymerase chain reaction assay from nasopharyngeal sampling at admission; 2) History or physical examination suggesting chronic lung disease, congenital heart disease, nephrotic syndrome, inflammatory disorders (systemic lupus erythematosus, ulcerative colitis), tumor, vascular malformation, trauma or recent major surgery; 3) Presence of a central venous catheter, immobilization for ≥ 72 h, or use of contraceptives; 4) Failure to perform CTPA; 5) Patients’ guardians unable or unwilling to provide written informed consent.

The definition of suspected PE and the screening process were prespecified and standardized across all participating centers before study initiation. The same screening criteria and case report form were used at all centers, and CTPA was performed to confirm or exclude PE. The demographic and clinical characteristics of the patients were recorded. Peripheral blood samples were obtained to measure inflammatory markers, coagulation function, liver function, and autoimmune antibody levels. The inflammatory markers included complete blood cell count, CRP, lactate dehydrogenase (LDH), ferritin, and interleukin-6 (IL-6). Composite inflammatory indices were calculated, including the neutrophil-to-lymphocyte ratio (NLR), platelet-to-lymphocyte ratio (PLR), systemic immune-inflammation index (SII), and systemic inflammation response index (SIRI) (22). These indices were calculated as follows: NLR = neutrophil count/lymphocyte count; PLR = platelet count/lymphocyte count; SII = platelet count  ×   neutrophil count/lymphocyte count; and SIRI = neutrophil count  ×   monocyte count/lymphocyte count. Autoimmune antibodies included LAC and ANA. The coagulation function included prothrombin time, activated partial thromboplastin time, thrombin time, D-dimer level, fibrinogen level, and thromboelastography. When PE was confirmed, the severity and risk of early death were assessed using hemodynamic stability, simplified Pulmonary Embolism Severity Index, right ventricular function, and cardiac biomarkers (14). Doppler ultrasonography was performed to examine the presence of deep venous thrombosis (DVT). Additionally, patients with confirmed PE underwent further thrombophilia and autoimmunity screening as part of the clinical evaluation, including antithrombin III, protein C, protein S, detailed autoantibodies, anticardiolipin (aCL) antibodies, and anti-*β*2-glycoprotein I (a*β*2GPI) antibodies. These additional tests were not routinely performed in patients without PE.

Following an initial assessment, patients were administered intravenous macrolides, tetracycline, quinolone, corticosteroids, immunoglobulin, heparin, or oxygen supplements based on the clinical guidelines and the individual patient’s medical status. Throughout hospitalization, a range of laboratory tests, including complete blood cell counts, inflammatory markers, liver function, and coagulation function, were regularly monitored as appropriate.

### 2.2 Definitions

RMPP was diagnosed according to the Chinese guideline for the diagnosis and treatment of MPP in children (3). Briefly, RMPP was defined as MPP with persistent fever or worsening pulmonary radiological findings despite standard macrolide treatment for 7 days or longer. MPP was diagnosed based on clinical manifestations consistent with M. pneumoniae infection and laboratory evidence of M. pneumoniae infection, including positive serologic testing for M. pneumoniae immunoglobulin M (IgM) antibody or positive M. pneumoniae RNA detected in nasopharyngeal swabs or sputum samples. M. pneumoniae IgM was detected using an assay (ELISA) kit (Oumeng, Beijing, China). M. pneumoniae RNA was detected with quantitative real-time polymerase chain reaction using M. pneumoniae simultaneous ampliﬁcation and testing kit (Rendu Biotechnology, Shanghai, China).

The diagnostic criteria for PE on CTPA encompassed the identification of a sharply demarcated complete or partial filling defect within the pulmonary arteries observable on a minimum of two consecutive images (14). Two thoracic radiologists independently evaluated CTPA findings, and a third radiologist adjudicated discordant results. The location of the PE was categorized as the main pulmonary artery, lobar branches, segmental branches, or subsegmental branches.

ANA were tested by a multiplexed bead immunoassay using a BioPlex 2200 system ANA screen kit (Bio-Rad, Hercules, California, USA), adhering to the manufacturer’s standard protocols, and an ANA titer ≥ 1:80 was deemed positive. LAC was detected by a dilute Russell’s viper venom time (dRVVT)-based assay using the HemosIL dRVVT screen, and dRVVT confirm reagents (Instrumentation Laboratory, Bedford, Massachusetts, USA) according to the International Society on Thrombosis and Haemostasis recommendations (23). LAC was considered positive if the normalized dRVVT ratio (screen/confirmation ratio) exceeded 1.2.

### 2.3 Statistical analysis

Data distributions were assessed using the Shapiro–Wilk test. Continuous variables were presented as mean ± standard deviation or median with interquartile range (IQR), and categorical variables as frequencies and percentages. Between-group comparisons were performed using Student’s t test, Mann–Whitney U test, *χ*2 test, or Fisher’s exact test, as appropriate. Admission D-dimer was transformed as log2(D-dimer/500 μg/L) to express its elevation relative to the prespecified clinical threshold and to reduce skewness. Univariate and multivariate logistic regression analyses were performed to identify factors associated with PE. Candidate variables for the multivariate model were selected according to clinical relevance and/or a univariate *P*-value ≤ 0.10. To reduce overfitting and improve model stability, variables with substantial clinical overlap were not entered simultaneously, and the final model was fitted using the enter method. Multicollinearity was assessed using variance inflation factors. Model discrimination was evaluated using the area under the receiver operating characteristic curve, and calibration was assessed using the Hosmer–Lemeshow test and Brier score. Results were reported as odds ratios (ORs) with 95% confidence intervals (CIs). A two-sided *P*-value < 0.05 was considered statistically significant.

## 3 Results

### 3.1 Patients and clinical characteristics

**Figure 1:**
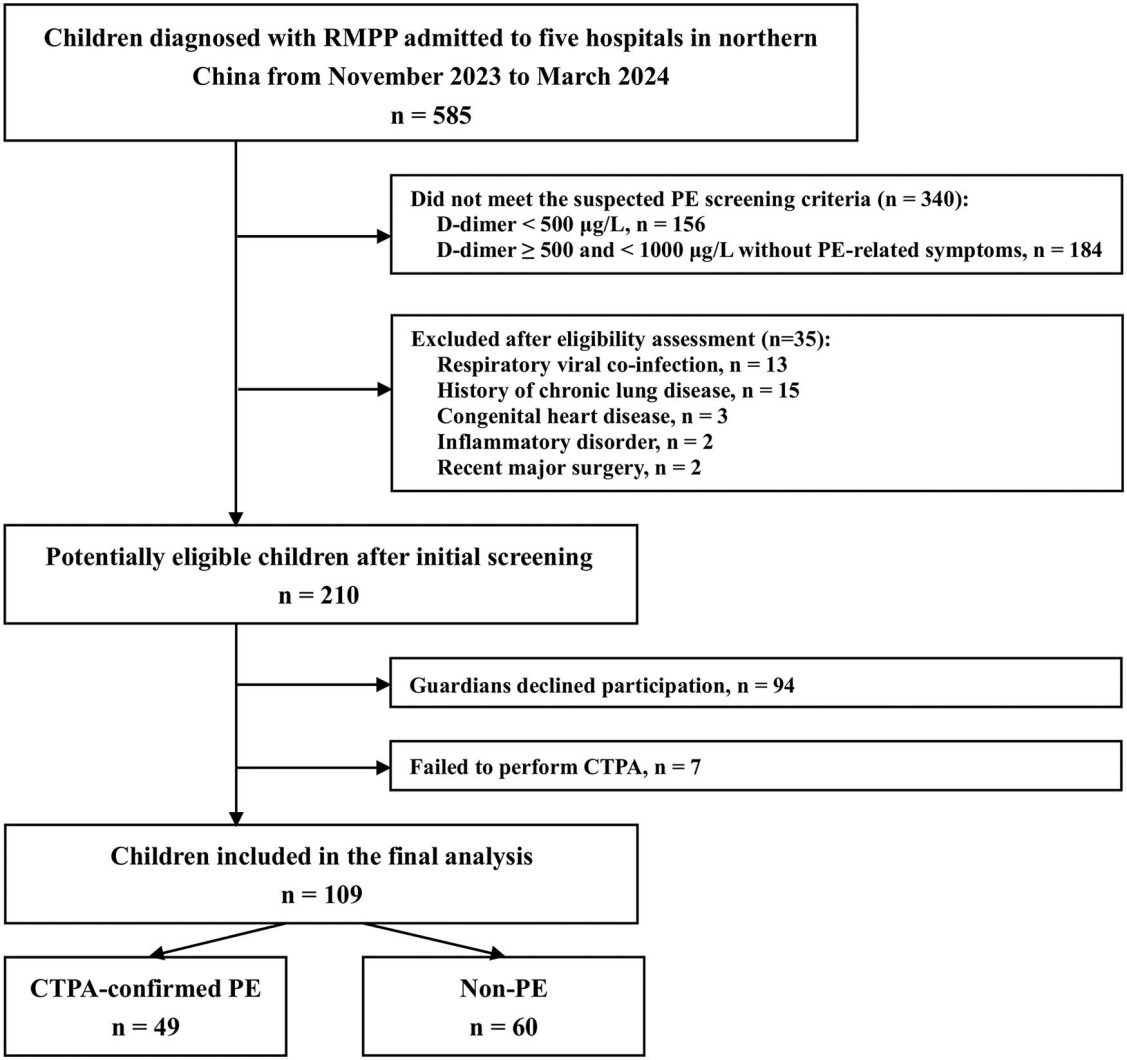
Flow chart of enrolled participants.

**Figure 2:**
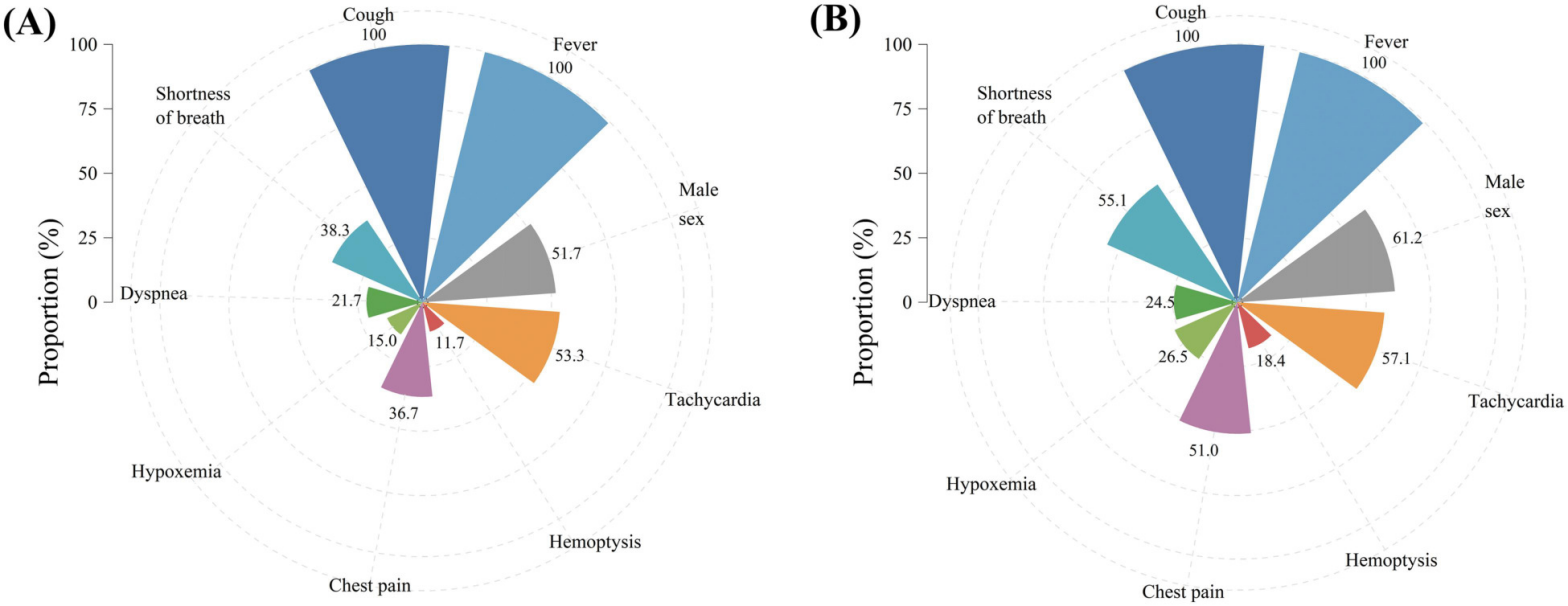
Polar bar plots comparing clinical characteristics between RMPP patients with and without PE. **(A)** patients without PE and **(B)** patients with PE. RMPP, refractory *mycoplasma pneumoniae* pneumonia; PE, pulmonary embolism.

Overall, 109 patients with RMPP and elevated D-dimer levels from five hospitals in northern China participated in this study (Figure 1). Among these patients, 49 (45.0%) were diagnosed with PE. Table 1 presents the demographic and clinical characteristics of the study participants. The mean age was 8.4 ± 2.3 years; 61 (60.0%) were boys and 48 (40.0%) were girls. The patients were admitted to the hospital at a median interval of 12.0 (IQR, 8.0–17.0) days after disease onset. As shown in Figure 2, fever and cough were the predominant symptoms. Additionally, clinical signs and symptoms suggestive of PE were observed, such as tachycardia (55.0%), shortness of breath (45.9%), chest pain (43.1%), dyspnea (22.9%), hypoxemia (20.2%), and hemoptysis (14.7%), which did not differ in the prevalence between PE and non-PE groups. Among the subset of patients experiencing chest pain (*n* = 47), the most common localization was in the intercostal region on the same side as the pleural effusion, and exacerbation occurred during deep breathing or coughing. The treatment regimens received by the participants before admission did not differ between the two groups (Supplementary file 1: Supplementary Table 1). Sixteen (14.7%) patients received intravenous corticosteroid therapy due to excessive inflammation, and 13 (11.9%) patients received heparin systemic anticoagulation due to elevated D-dimer levels.

**Table 1:** Clinical characteristics of all patients with RMPP and suspected PE.

Characteristics	Total (*n* = 109)	Non-PE (*n* = 60)	PE (*n* = 49)	*P*-value
Male sex	61 (60.0%)	31 (51.7%)	30 (61.2%)	0.339
Age (years)	8.4 ± 2.3	8.8 ± 2.3	8.0 ± 2.2	0.102
BMI (kg/m^2^)	16.4 (14.7–20.5)	15.9 (14.7–20.0)	17.6 (14.6–21.3)	0.536
Duration from onset to admission (days)	12.0 (8.0–17.0)	12.0 (8.0–19.0)	12.0 (9.0–17.0)	0.869
Maximum body temperature (℃)	40.0 (39.5–40.2)	40.0 (39.7–40.3)	40.0 (39.5–40.0)	0.088
Heart rate (beats/min)	122.5 ± 15.9	121.0 ± 14.4	124.4 ± 17.4	0.289
Tachycardia	60 (55.0%)	32 (53.3%)	28 (57.1%)	0.704
Respiratory rate (beats/min)	28.0 (26.0–32.0)	27.0 (26.0–32.0)	30.0 (25.0–32.0)	0.541
Shortness of breath	50 (45.9%)	23 (38.3%)	27 (55.1%)	0.087
Dyspnea	25 (22.9%)	13 (21.7%)	12 (24.5%)	0.820
Hypoxemia	22 (20.2%)	9 (15.0%)	13 (26.5%)	0.156
Location of chest pain	47 (43.1%)	22 (36.7%)	25 (51.0%)	0.174
Consistent with pleural effusion	20 (42.6%)	8 (36.4%)	12 (48.0%)	0.556
Intercostal	23 (48.9%)	11 (50.0%)	12 (48.0%)	1.000
Back	7 (14.9%)	2 (9.1%)	5 (20.0%)	0.423
Subxiphoid	2 (4.3%)	2 (9.1%)	0 (0.0%)	0.214
Shoulder	3 (6.4%)	1 (4.5%)	2 (8.0%)	1.000
Aggravating factors for chest pain				
Deep breathing or coughing	17 (36.2%)	9 (40.9%)	8 (32.0%)	0.558
Change in body position	9 (23.7%)	4 (22.2%)	5 (25.0%)	1.000
Hemoptysis	16 (14.7%)	7 (11.7%)	9 (18.4%)	0.417

Data are expressed as mean ± SD, median (IQR) or *n* (%) and compared between groups using the Student's *t* test, Mann−Whitney *U* test, or *χ*^2^ test (or Fisher's exact test).

RMPP, refractory *mycoplasma pneumoniae* pneumonia; PE, pulmonary embolism; BMI, body mass index.

### 3.2 Inflammatory features

Children with PE had higher white blood cell and neutrophil counts than those without PE (*P* < 0.05), whereas lymphocyte and platelet counts were comparable between groups. Composite inflammatory indices derived from routine blood cell counts were further evaluated. SIRI was higher in children with PE than in those without PE (*P* = 0.019), whereas NLR, PLR, and SII did not differ significantly between groups. Several inflammatory and coagulation-related markers, including CRP, LDH, ferritin, IL-6, and D-dimer, were also assessed. CRP, ferritin, LDH, and IL-6 levels at admission (Supplementary file 1: Supplementary Table 2) and their peak levels (Table 2) did not differ significantly between the PE and non-PE groups (*P* > 0.05). By contrast, D-dimer levels were higher in the PE group both at admission (3005.0 μg/L vs. 1974.0 μg/L; *P* < 0.001) and at peak level (4660.0 μg/L vs. 2976.0 μg/L; *P* = 0.003). When expressed relative to the prespecified threshold of 500 μg/L, the admission D-dimer level was approximately 6.01-fold in the PE group and 3.95-fold in the non-PE group (*P* < 0.001). Patients with PE also had higher ratios of D-dimer max/LDH max (7.0 vs. 4.6; *P* = 0.001) and D-dimer max/ferritin max (12.1 vs. 7.8; *P* = 0.026), indicating a greater increase in D-dimer relative to LDH and ferritin in the PE group (Supplementary file 1: Supplementary Table 2).

**Table 2:** Inflammatory and immunologic features of all patients with RMPP and suspected PE.

Characteristics	Total (*n* = 109)	Non-PE (*n* = 60)	PE (*n* = 49)	*P*-value
Complete blood cell count at admission				
White blood cell count (10^9^/L)	9.4 (7.1–11.9)	9.2 (6.3–11.2)	10.2 (8.1–14.0)	0.023
Neutrophil (%)	77.7 (70.4–85.5)	76.0 (68.8–86.4)	78.5 (73.1–85.4)	0.404
Neutrophil count (10^9^/L)	7.6 (5.3–9.4)	7.0 (4.6–8.6)	8.0 (6.2–10.9)	0.021
Lymphocyte (%)	11.7 (7.8–19.6)	12.7 (7.8–21.2)	11.4 (7.6–17.1)	0.446
Lymphocyte count (10^9^/L)	1.1 (0.8–1.7)	1.1 (0.7–1.6)	1.1 (0.8–1.9)	0.483
Platelet count (10^9^/L)	279.0 (218.0–370.0)	282.0 (226.0–370.8)	268.0 (207.0–370.0)	0.611
NLR	6.55 (3.63–11.67)	5.96 (3.25–11.89)	6.91 (4.47–10.86)	0.390
PLR	243.75 (176.36–371.25)	254.41 (174.77–396.00)	241.82 (180.00–364.44)	0.413
SII	1980.00 (973.88–3360.00)	1941.75 (842.47–3076.62)	2145.00 (1170.25–3381.75)	0.382
SIRI	4.65 (2.07–8.71)	3.76 (1.66–6.84)	5.11 (3.03–11.32)	0.019
CRP max (mg/L)	60.6 (33.8–115.7)	57.3 (31.0–121.9)	69.0 (41.0–115.5)	0.422
LDH max (IU/L)	627.0 (471.0–788.9)	625.0 (467.1–797.7)	642.0 (490.5–784.5)	0.951
Ferritin max (ng/mL)	347.1 (199.8–701.4)	342.7 (175.2–713.2)	357.1 (213.8–662.7)	0.848
IL-6 max (pg/mL)	22.2 (6.9–67.6)	15.7 (7.1–59.3)	36.7 (5.6–75.4)	0.363
D-dimer at admission (*μ*g/L)	2484.0 (1352.5–3455.0)	1974.0 (1075.0–3072.3)	3005.0 (2208.0–4520.5)	<0.001
Admission D-dimer/500 μg/L, fold	4.97 (2.71–6.91)	3.95 (2.17–6.12)	6.01 (4.46–8.96)	<0.001
D-dimer max (μg/L)	3363.0 (2267.5–5906.0)	2976.0 (1830.0–4859.0)	4660.0 (3145.5–7300.0)	0.003
Procalcitonin max (ng/mL)	0.23 (0.12–0.67)	0.25 (0.14–0.66)	0.22 (0.10–0.74)	0.535
ALT max (IU/L)	73.0 (39.5–123.5)	75.5 (39.0–124.3)	65.0 (40.5–126.0)	0.805
Albumin nadir (g/L)	31.7 ± 3.7	32.2 ± 3.7	31.1 ± 3.7	0.120
D-dimer max/CRP max ratio	56.6 (27.0–120.1)	48.7 (25.1–113.0)	63.4 (34.8–129.0)	0.139
D-dimer max/LDH max ratio	5.8 (3.4–8.6)	4.6 (2.8–7.0)	7.0 (4.7–9.6)	0.001
D-dimer max/Ferritin max ratio	10.3 (5.2–16.8)	7.8 (4.6–14.9)	12.1 (7.4–20.7)	0.026
D-dimer max/IL-6 max ratio	187.4 (50.0–534.1)	198.9 (41.4–473.0)	154.5 (51.0–750.0)	0.668
Thromboelastography	82/103 (79.6%)	48/56 (85.7%)	34/47 (72.3%)	0.140
Positive LAC	64 (58.7%)	30 (50.0%)	34 (69.4%)	0.051
Positive ANA	76 (69.7%)	37 (61.7%)	39 (79.6%)	0.059
Simultaneously positive LAC and ANA	48 (44.0%)	19 (31.7%)	29 (59.2%)	0.006

Data are expressed as mean ± SD, median (IQR) or *n* (%) and compared between groups using the Student's *t* test, Mann−Whitney *U* test, or *χ*^2^ test (or Fisher's exact test).

RMPP, refractory *mycoplasma pneumoniae* pneumonia; PE, pulmonary embolism; NLR, neutrophil-to-lymphocyte ratio; PLR, platelet-to-lymphocyte ratio; SII, systemic immune-inflammation index; SIRI, systemic inflammation response index; CRP, C-reactive protein; LDH, lactate dehydrogenase; IL-6, interleukin-6; ALT, alanine aminotransferase; LAC, lupus anticoagulant; ANA, antinuclear antibodies.

### 3.3 Immunologic investigations

As shown in Table 2, LAC and ANA were detected in 64 (58.7%) and 76 (69.7%) patients, respectively. The positivity rates for LAC (69.4% vs. 50.0%; *P* = 0.051) and ANA (79.6% vs. 61.7%; *P* = 0.059) tended to be higher in patients with PE compared to those without. Notably, simultaneous positivity for LAC and ANA was more frequent in patients with PE (59.2% vs. 31.7%; *P* = 0.006). Because further thrombophilia and autoimmunity screening was performed only in patients with confirmed PE, the following results are presented descriptively. Among the 49 patients with PE, nine patients (18.4%) were positive for additional autoantibodies, including seven positive for anti-RNP-A, one positive for anti-Ro/SSA, and one positive for anti-centromere antibodies. All 49 children tested negative for anti-Smith, anti-double-stranded DNA, and antineutrophil cytoplasmic antibodies. Ten patients (20.4%) were positive for aCL antibodies, including five for IgM, four for IgG, and one for IgA. a*β*2GPI antibodies were positive in four patients, including dual IgM and IgG positivity in one patient, isolated IgM positivity in two patients, and isolated IgG positivity in one patient. Protein S activity was low in 6.1% (3/49) of patients, while protein C activity was normal. Antithrombin Ⅲ activity was low in 4.1% (2/49) of the patients.

### 3.4 Radiographic and bronchoscopy features

Pulmonary consolidation was found in all patients, with the right (50.5%) and left (40.4%) lower lobes being the most commonly affected (Table 3). Compared with patients without PE, those with PE had a significantly higher rate of left lower lobe consolidation (57.1% vs. 26.7%; *P* = 0.002) and bilateral consolidation (28.6% vs. 13.3%; *P* = 0.049). The incidences of necrotizing pneumonia and pleural effusion did not differ significantly between the two groups (*P* = 0.265). Among the 70 patients who underwent bronchoscopy, mucosal inflammation (91.4%) and mucous plugs (60.0%) were the most common macroscopic findings, with neutrophilic inflammation demonstrated by bronchoalveolar lavage fluid differential cell counts (Table 3). Notably, three patients with PE had bloody bronchoalveolar lavage fluid from the lobes without consolidation.

**Table 3:** Radiography and bronchoscopy findings of all patients with RMPP and suspected PE.

Findings	Total (*n* = 109)	Non-PE (*n* = 60)	PE (*n* = 49)	*P*-value
Consolidation				
Left upper lobe	22 (20.2%)	12 (20.0%)	10 (20.4%)	1.000
Left lower lobe	44 (40.4%)	16 (26.7%)	28 (57.1%)	0.002
Right upper lobe	22 (20.2%)	14 (23.3%)	8 (16.3%)	0.473
Right middle lobe	25 (22.9%)	17 (28.3%)	8 (16.3%)	0.172
Right lower lobe	55 (50.5%)	35 (58.3%)	20 (40.8%)	0.084
More than 2 lobes	44 (40.4%)	23 (38.3%)	21 (42.9%)	0.632
Bilateral	22 (20.2%)	8 (13.3%)	14 (28.6%)	0.049
Both lower lobes	10 (9.2%)	2 (3.3%)	8 (16.3%)	0.041
Pleural effusion	63 (57.8%)	32 (53.3%)	31 (63.3%)	0.334
Necrotizing pneumonia	27 (24.8%)	12 (20.0%)	15 (30.6%)	0.265
Bronchoscopy findings	*n* = 70	*n* = 36	*n* = 34	
Macroscopic findings				
Mucosal edema and hyperemia	64 (91.4%)	33 (91.7%)	31 (91.2%)	1.000
Mucous plug	42 (60.0%)	22 (61.1%)	20 (58.8%)	1.000
Mucosal necrosis	11 (15.7%)	8 (22.2%)	3 (8.8%)	0.190
Plastic bronchitis	4 (5.7%)	2 (5.6%)	2 (5.9%)	1.000
BAL fluid differential cell counts				
Neutrophil (%)	60.5 (43.5–76.8)	67.0 (39.0–76.8)	58.0 (44.0–75.8)	0.716
Macrophage (%)	20.5 (9.0–40.0)	20.0 (9.0–41.5)	21.0 (8.5–38.3)	0.953
Lymphocyte (%)	10.5 (6.3–17.8)	9.0 (6.0–14.8)	13.5 (8.0–20.5)	0.161

Data are expressed as median (IQR) or *n* (%) and compared between groups using the Mann−Whitney *U* test or *χ*^2^ test (or Fisher's exact test).

RMPP, refractory *mycoplasma pneumoniae* pneumonia; PE, pulmonary embolism.

**Figure 3:**
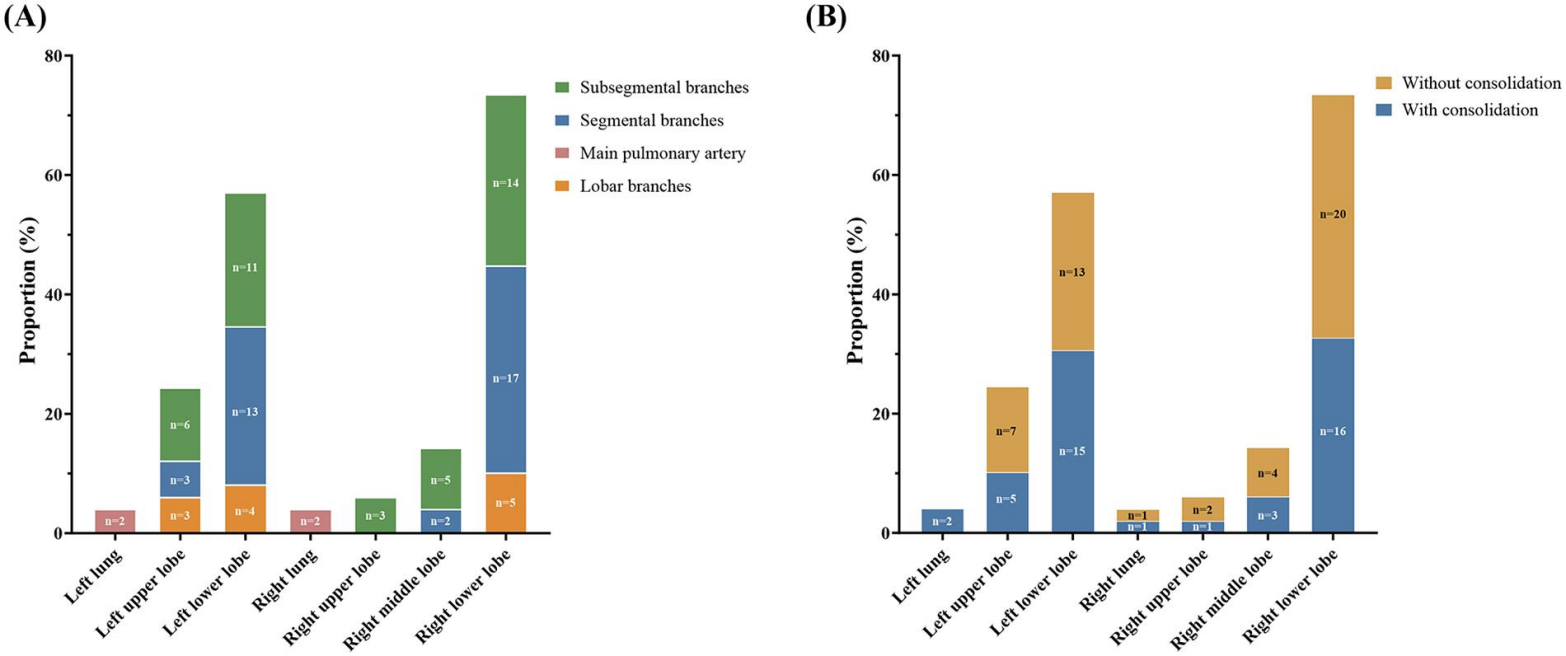
Bar graph shows the anatomic distribution of 90 pulmonary emboli in 49 CTPA studies that were positive for PE. **(A)** pulmonary artery location and **(B)** association with consolidation. CTPA, computed tomographic pulmonary angiography; PE, pulmonary embolism.

Among the 49 patients with PE, 90 pulmonary emboli were identified; their anatomical locations are shown in Figure 3A. PE was observed in more than one lobe in 25 patients (51.0%) and bilaterally in 24 patients (49%). Of the 90 pulmonary emboli, 47 (52.2%) occurred in lobes without consolidation (Figure 3B). DVT evaluations showed that five patients experienced extrapulmonary embolism: four with cardiac embolism and one with cerebral embolism.

### 3.5 Factors associated with PE in children with RMPP and elevated D-dimer

Univariate and multivariate logistic regression analyses were conducted to identify factors associated with PE (Table 4). In multivariate logistic regression analysis, neutrophil count, log2(admission D-dimer/500 μg/L), simultaneous positivity for LAC and ANA, and left lower lobe consolidation were independently associated with CTPA-confirmed PE. SIRI and the D-dimer max/LDH max ratio were also entered into the multivariate model but were not independently associated with PE after adjustment. The variance inflation factors ranged from 1.042 to 2.140, indicating no apparent multicollinearity. The model showed acceptable discrimination and calibration, with an AUC of 0.839, a Brier score of 0.164, and a Hosmer–Lemeshow *P*-value of 0.792.

**Table 4:** Factors associated with PE in patients with RMPP and elevated D-dimer.

Factors		Univariate analysis		Multivariate analysis	
		OR (95% CI)	*P*-value	OR (95% CI)	*P*-value
Neutrophil count	Per 10^9^/L	1.157 (1.037–1.291)	0.009	1.221 (1.031–1.446)	0.021
SIRI	Per 1	1.092 (1.011–1.180)	0.026	0.948 (0.836–1.075)	0.403
Log_2_(admission D-dimer/500 μg/L)	Per doubling	2.412 (1.499–3.883)	<0.001	2.664 (1.485–4.778)	0.001
Log_2_(peak D-dimer/500 μg/L)	Per doubling	1.667 (1.149–2.419)	0.007		
D-dimer max/ferritin max ratio	Per 1	1.016 (0.994–1.038)	0.167		
D-dimer max/LDH max ratio	Per 1	1.084 (1.009–1.166)	0.028	1.018 (0.949–1.092)	0.612
Simultaneously positive LAC and ANA	No	1 (ref)			
	Yes	3.129 (1.423–6.878)	0.005	3.185 (1.234–8.223)	0.017
Left lower lobe consolidation	No	1 (ref)			
	Yes	3.667 (1.640–8.199)	0.002	5.016 (1.825–13.790)	0.002
Bilateral consolidation	No	1 (ref)			
	Yes	2.600 (0.987–6.849)	0.053		
Both lower lobes consolidation	No	1 (ref)			
	Yes	5.659 (1.142–28.034)	0.034		

RMPP, refractory *mycoplasma pneumoniae* pneumonia; PE, pulmonary embolism; OR, odds ratio; CI, confidence interval; SIRI, systemic inflammation response index; LDH, lactate dehydrogenase; LAC, lupus anticoagulant; ANA, antinuclear antibodies.

## 4 Discussion

This prospective multicenter observational study assessed clinical characteristics and factors associated with PE in children admitted for RMPP. These results suggest that children with RMPP who develop PE may present with symptoms such as tachycardia, shortness of breath, chest pain, dyspnea, hypoxemia, and hemoptysis but without specificity. Inflammatory markers (CRP, LDH, ferritin, and IL-6) previously linked to PE caused by MPP (24) were not significantly associated with PE when only children with RMPP were analyzed. Patients with RMPP and PE exhibited significantly elevated neutrophil counts, D-dimer levels, rates of simultaneously positive LAC and ANA, and rates of left lower lobe consolidation compared to those without PE.

Plasma levels of D-dimer, a breakdown product of cross-linked fibrin, are elevated in the presence of acute thrombosis. D-dimer testing has been recommended as a diagnostic strategy for ruling out PE due to its high negative predictive value (14–16). Kanis et al. (15) suggested that a normal D-dimer level could reliably exclude PE in pediatric patients with a low clinical probability. Consequently, patients with RMPP who exhibited normal D-dimer levels were excluded. However, the positive predictive value of D-dimer was low, particularly in patients with severe infections such as RMPP. D-dimer, neutrophil, CRP, LDH, ferritin, and IL-6 have been identified as markers of excessive inflammatory response and factors associated with RMPP (17, 25–27). In addition to individual blood cell counts, composite inflammatory indices may provide further information on infection-related systemic inflammation. In our study, SIRI was higher in children with PE, whereas NLR, PLR, and SII did not differ significantly between groups. However, SIRI was not independently associated with PE after adjustment, suggesting that it may reflect infection-related inflammatory severity but did not provide additional independent information beyond neutrophil count and other clinical variables in this cohort. M. pneumoniae infection can cause microvascular endothelial injury due to cytokines and chemokines, leading to local vasculitis without systemic hypercoagulability (28, 29). Vasculitis activates coagulation and secondary fibrinolysis, resulting in production of D-dimer (18, 27). Coagulation and fibrinolytic systems are in dynamic equilibrium, and no macroscopic blood clots occur (30, 31). Therefore, patients could exhibit elevated D-dimer levels without thrombosis. However, because elevated D-dimer was part of the inclusion criteria, its association with PE should be interpreted cautiously. In this selected high-risk cohort, the admission D-dimer level, rather than the peak D-dimer level, was associated with CTPA-confirmed PE. Once thrombosis occurs, the fibrinolytic system is activated, which results in excessively elevated D-dimer levels. A study involving 43 children with SMPP complicated by thrombosis revealed that D-dimer levels peaked within 6–15 days of disease onset (5). In our study, the median duration from disease onset to admission was 12.0 days, and these findings suggest that peak D-dimer levels may have occurred before or at admission in some patients. After the excessive inflammatory response is controlled, vascular injury and coagulation activation may gradually improve, leading to a decrease in D-dimer levels. The absorption of PE in children requires 1–3 months or longer (5, 9). Therefore, dynamic monitoring of D-dimer levels is crucial in children with RMPP and suspected PE.

In our cohort, LAC and ANA positivity was common overall and was also present in many children without PE, indicating that these antibodies are not specific to PE. In acute M. pneumoniae infection, such findings may represent transient infection-related immune responses. Previous reports have described transient LAC and ANA positivity in patients with M. pneumoniae infection and thrombosis (5, 9, 24), and molecular mimicry between M. pneumoniae components and host cell structures may contribute to autoantibody formation (28, 32). Evidence from autoimmune and thrombotic disorders has linked LAC and ANA to endothelial dysfunction, hypercoagulability, impaired fibrinolysis, and thrombosis (20, 21, 33–38), which provides a plausible biological background for the higher frequency of double positivity observed in children with PE. Nevertheless, this finding may also reflect more intense infection-related immune activation or systemic inflammation rather than a direct pathogenic effect of these antibodies. In our study, neither LAC nor ANA positivity alone was independently related to PE, whereas simultaneous positivity for both antibodies was associated with CTPA-confirmed PE. Thus, LAC and ANA double positivity should be viewed as an immune-related feature in this selected high-risk RMPP cohort. Because functional experiments were not performed, causality cannot be inferred from the present study. Further studies with serial antibody testing and mechanistic validation are needed to determine whether these antibodies participate in thrombosis or simply reflect the inflammatory and immune status of children with RMPP complicated by PE.

The origin of PE in MPP cannot be fully elucidated at present, but evidence suggests the *in situ* formation of a pulmonary artery thrombus rather than a more traditional embolic event originating from DVT. Vasculitis induced by M. pneumoniae has been documented in various organs, including the skin, lung, heart, and brain (39–41), while thrombosis in MPP appears to predominantly involve the pulmonary artery (5). This pattern suggests that pulmonary thrombosis may be related to vascular endothelial injury and adjacent tissue inflammation in the lung. Lobar consolidation, particularly involving ≥ 2/3 of a lobe, was found to be associated with PE in MPP (5, 9, 12). Our study further revealed that consolidation of the left lower lobe was independently associated with PE. In cases of MPP, the right lung is slightly more frequently affected than the left lung, particularly the right lower lobe (42). Although the right and left lower lobes were affected at similar rates in our cohort of children with RMPP, the left lower lobe was more commonly affected in patients with PE than in those without PE (57.1% vs. 26.7%), making it a significant feature. Lobar consolidation or lower-lobe involvement has also been described in previous studies and case reports of MPP-associated PE (6, 9, 19). In our study, bilateral consolidation and both lower lobe consolidations were more common in patients with PE than in those without PE. However, these were not independently associated with PE in the univariate and multivariate analyses. Thus, it remains unclear why left lower lobe consolidation is associated with the occurrence of PE. One possible explanation is that left lower lobe consolidation may represent a marker of more severe regional pulmonary involvement. Previous studies have shown that PE in children with MPP and pulmonary consolidation tends to occur in patients with more severe clinical and inflammatory features, such as longer fever duration and higher D-dimer levels (9, 43). However, the anatomical specificity of the left lower lobe remains uncertain. In our cohort, approximately half of the emboli occurred in lobes without consolidation, suggesting that thrombi do not necessarily develop within the consolidated lobe. This is consistent with previous reports showing discordance between the location of consolidation and pulmonary thrombi (9). Therefore, this association may reflect regional pulmonary inflammation, endothelial injury, and systemic inflammation-coagulation activation rather than a direct location-specific effect. Further studies with larger cohorts and detailed lobar-level imaging analyses are needed to clarify this finding.

Necrotizing pneumonia is a potential complication of PE caused by M. pneumoniae (5, 11). The reported incidence of necrotizing pneumonia in patients with MPP and PE ranges from 12.5%–59.2% (5, 6, 24). In our study, 15 patients (30.6%) in the PE group developed necrotizing pneumonia. Necrotizing pneumonia may be related to pulmonary infarction caused by intrapulmonary vascular occlusion (5). However, our findings indicate a similar incidence of necrotizing pneumonia in patients with and without PE, which is consistent with the results of another study (9). In addition, a study by Fu et al. revealed that necrotizing pneumonia occurred in lobes without embolisms (44). Therefore, thrombotic occlusion of the large vessels may not be responsible for necrotizing pneumonia. Another study evaluating the risk factors for necrotizing pneumonia caused by M. pneumoniae observed decreased enhancement areas on contrast-enhanced chest CT images in the early stages and found that the use of low-molecular-weight heparin reduced the risk of pulmonary necrosis (43). Further studies are required to determine the potential involvement of microvascular thrombosis in the development of necrotizing pneumonia.

This study has some limitations. First, only children with RMPP, elevated D-dimer, clinical suspicion of PE, and completed CTPA were included. Therefore, the non-PE group did not represent the general RMPP population, and selection bias could not be excluded. However, the aim of this study was to explore factors associated with PE among children with elevated D-dimer levels. In clinical practice, CTPA is generally performed only when PE is suspected rather than as a routine examination. Therefore, the findings should be interpreted as associations within this selected high-risk cohort and should not be extrapolated to all children with RMPP. Second, aCL and a*β*2GPI antibodies were not measured in patients without PE. Thus, these results were descriptive and could not be compared between groups. Future studies should assess a standardized antiphospholipid antibody panel in both PE and non-PE patients. Third, the sample size was relatively small, and the number of PE events was limited. Although model diagnostics were performed, the identified associations require validation in larger cohorts.

## 5 Conclusions

This study identified several factors associated with CTPA-confirmed PE among hospitalized children with RMPP, elevated D-dimer levels, and clinical suspicion of PE. Elevated neutrophil counts, greater admission D-dimer elevation, simultaneous positivity for LAC and ANA, and left lower lobe consolidation were associated with PE, whereas CRP, LDH, ferritin, and IL-6 were not significantly associated with PE in this selected cohort.

## Data Availability

The original contributions presented in the study are included in the article/Supplementary Material, further inquiries can be directed to the corresponding author.
