# Supplementary material for: Factors associated with pulmonary embolism in children with refractory Mycoplasma pneumoniae pneumonia and elevated D-dimer
Source: Front Pediatr. 2026 Jun 25;14:1847107. doi: 10.3389/fped.2026.1847107 (PMC13345890; doi:10.3389/fped.2026.1847107)
Supplement: Supplementary file 1 [file Table1.docx]

**Supplementary Information**

Supplementary file 1: Table S1 Treatments before and after admission. Table S2 Inflammatory and immunologic features of all patients with RMPP and suspected PE.

**Table S1** Treatments before and after admission.

| **Characteristics** | **Total**  **(n = 109)** | **Non-PE**  **(n = 60)** | **PE**  **(n = 49)** | ***P*-value** |
| --- | --- | --- | --- | --- |
| Corticosteroid therapy (before admission) | 16 (14.7%) | 7 (11.7%) | 9 (18.4%) | 0.417 |
| Maximum dose (mg/kg/day) | 2.0 (2.0−4.0) | 2.0 (1.6−3.6) | 2.7 (2.0−4.0) | 0.110 |
| Duration (days) | 5.0 (3.0−7.0) | 5.0 (2.5−6.5) | 5.0 (4.0−7.8) | 0.130 |
| Macrolides (before admission) | 109 (100%) | 60 (100%) | 49 (100%) | 1.000 |
| Duration (days) | 7.0 (5.0−10.0) | 7.0 (5.0−10.0) | 8.0 (5.0−10.0) | 0.140 |
| Heparin (before admission) | 13 (11.9%) | 7 (11.7%) | 6 (12.2%) | 1.000 |
| Duration (days) | 2.0 (1.0−5.0) | 2.0 (1.0−5.0) | 2.0 (1.0−3.8) | 0.731 |
| Oxygen supplement (after admission) | 51 (46.8%) | 24 (40.0%) | 27 (55.1%) | 0.127 |
| Maximum flow (L/min) | 3.0 (2.0−3.0) | 2.0 (1.0−3.8) | 1.5 (1.0−3.0) | 0.441 |
| Duration (days) | 6.4 ± 3.4 | 6.4 ± 2.7 | 6.3 ± 3.9 | 0.965 |
| Corticosteroid therapy (after admission) | 99 (90.8%) | 54 (90.0%) | 45 (91.8%) | 1.000 |
| Maximum dose (mg/kg/day) | 4.0 (3.6−6.0) | 4.0 (4.0−6.2) | 4.0 (3.0−6.3) | 0.319 |
| Duration (days) | 9.0 (7.0−12.0) | 8.0 (6.8−10.3) | 10.0 (7.5−14.0) | 0.013 |
| IVIG (after admission) | 8 (7.3%) | 1 (1.7%) | 7 (14.3%) | 0.021 |
| Heparin (prior to CTPA) | 44 (40.4%) | 18 (30.0%) | 26 (53.1%) | 0.019 |
| Heparin (after admission) | 76 (69.7%) | 30 (50.0%) | 46 (93.9%) | <0.001 |
| Tetracycline or quinolone treatment (after admission) | 66 (60.6%) | 37 (61.7%) | 29 (59.2%) | 0.845 |
| Oral corticosteroid therapy (after discharge) | 86 (78.9%) | 42 (70.0%) | 44 (89.8%) | 0.017 |
| Length of hospital stay (days) | 10.0 (9.0−14.0) | 9.5 (7.0−11.0) | 12.0 (10.0−17.0) | <0.001 |

Data are expressed as mean ± SD, median (IQR) or n (%) and compared between groups using the Student's *t* test, Mann−Whitney *U* test, or χ^2^ test (or Fisher's exact test).

PE, pulmonary embolism; CTPA, computed tomographic pulmonary angiography.

**Table S2** Inflammatory and immunologic features of all patients with RMPP and suspected PE.

| **Characteristics** | **Total**  **(n = 109)** | **Non-PE**  **(n = 60)** | **PE**  **(n = 49)** | ***P*-value** |
| --- | --- | --- | --- | --- |
| CRP at admission (mg/L) | 42.7 (18.3–80.5) | 39.4 (16.2–78.3) | 43.9 (20.0–82.8) | 0.126 |
| LDH at admission (IU/L) | 578 (447.0–701.0) | 585.5 (390.75–669.8) | 575.0 (458.0–716.0) | 0.543 |
| Ferritin at admission (ng/mL) | 289.8 (191.6–555.9) | 294.3 (181.7–486.9) | 289.8 (192.1–565.6) | 0.674 |
| IL-6 at admission (pg/mL) | 17.8 (6.2–59.7) | 15.1 (6.75–48.8) | 23.9 (5.10–68.1) | 0.131 |
| Procalcitonin at admission (ng/mL) | 0.18 (0.10–0.53) | 0.19 (0.12–0.52) | 0.16 (0.09–0.53) | 0.509 |
| ALT at admission (IU/L) | 44.0 (26.0–86.0) | 40.5 (26.0–79.8) | 46.0 (27.0–97.0) | 0.744 |
| Albumin at admission (g/L) | 32.6 ± 3.9 | 33.0 ± 4.0 | 32.2 ± 3.7 | 0.302 |
| CRP max/IL-6 max ratio | 2.4 (1.2–10.4) | 4.0 (1.1–10.5) | 2.1 (1.2–10.5) | 0.513 |
| CRP max/Ferritin max ratio | 0.21 (0.11–0.33) | 0.18 (0.08–0.36) | 0.21 (0.12–0.32) | 0.713 |
| CRP max/LDH max ratio | 0.11 0.05–0.18) | 0.10 0.05–0.18) | 0.11 (0.06–0.18) | 0.503 |
| Ferritin max/LDH max ratio | 0.54 (0.33–1.04) | 0.50 (0.30–1.05) | 0.60 (0.36–0.88) | 0.609 |
| Ferritin max/IL-6 max ratio | 16.0 (4.8–52.2) | 23.2 (6.6–78.5) | 14.8 (4.6–45.2) | 0.344 |
| LDH max/IL-6 max ratio | 32.5 (9.1–92.3) | 43.4 (13.6–95.9) | 22.3 (8.8–82.3) | 0.288 |
| Immunoglobulins (g/L) | | | | |
| IgG | 9.4 (7.8–11.4) | 9.2 (7.8–10.6) | 9.8 (8.1–11.9) | 0.235 |
| IgA | 1.8 (1.2–2.3) | 1.7 (1.2–2.3) | 2.0 (1.3–2.4) | 0.315 |
| IgM | 2.3 (1.5–3.4) | 2.0 (1.4–3.4) | 2.6 (1.7–3.6) | 0.111 |
| Lymphocyte subsets (×10^6^/L) | n=92 | n=54 | n=38 |  |
| CD3 (total T cells) | 827.5 (538.5–1532.5) | 726.5 (510.0–1590.1) | 977.0 (556.8–1477.8) | 0.510 |
| CD4^+^/CD3^+^ (helper T cells) | 340.0 (241.5–810.8) | 332.0 (207.0–843.3) | 398.0 (257.8–802.3) | 0.549 |
| CD8^+^/CD3^+^ (cytotoxic T cells) | 415.5 (263.3–724.3) | 370.5 (261.8–728.0) | 465.5 (274.8–727.0) | 0.372 |
| CD19 (total B cells) | 235.0 (153.5–366.3) | 247.5 (145.3–396.0) | 220.0 (159.5–328.8) | 0.703 |
| CD56 and CD16 (NK cells) | 102.0 (70.0–150.8) | 96.5 (70.0–161.5) | 113.0 (60.5–147.8) | 0.516 |

Data are expressed as mean ± SD or median (IQR) and compared between groups using the Student's *t* test or Mann−Whitney *U* test.

RMPP, refractory *mycoplasma pneumoniae* pneumonia; PE, pulmonary embolism; CRP, C-reactive protein; LDH, lactate dehydrogenase; IL-6, interleukin-6; ALT, alanine aminotransferase; IgG, immunoglobulin G; IgA, immunoglobulin A; IgM, immunoglobulin M; NK cells, natural killer cells.
